# Emerging perspectives in respiratory allergic diseases: a review of future directions

**DOI:** 10.3389/falgy.2026.1819491

**Published:** 2026-04-29

**Authors:** Rafael Durán, Sergio E. Chiarella, Thanai Pongdee, Juyoung Choi, Martin Maillo, Cesar A. Galván

**Affiliations:** 1 Human Nutrition and Food Research Group (GINAH), Universidad Científica del Sur, Lima, Peru; 2 Emedic Salud, Lima, Peru; 3 Division of Allergic Diseases, Mayo Clinic, Rochester, MN, United States; 4 Facultad de Medicina “Alberto Hurtado”, Universidad Peruana Cayetano Heredia, Lima, Peru; 5 IBAMEDICA, Santa Fe, Argentina; 6 Clinical Allergy Service, Clínica Internacional, Lima, Peru

**Keywords:** allergic rhinitis, asthma, climate change, immunotherapy, precision medicine

## Abstract

**Background:**

Respiratory allergic diseases are experiencing changes influenced by genetic factors, environmental shifts, and social and demographic elements. These evolving patterns, together with rapid advances in diagnosis and treatment, require a thorough review of emerging trends.

**Objective:**

To examine future directions in managing respiratory allergic diseases, focusing on changing disease patterns, environmental factors, diagnostic innovations, and therapeutic advances toward precision medicine. A literature search was conducted in PubMed and Scopus databases covering 2015–2025. Studies addressing human populations with allergic rhinitis or asthma were included, emphasizing emerging patterns, environmental factors, diagnostic technologies, and therapeutic innovations, while reviews, conference proceedings, case reports, and studies without clinical relevance were excluded. Initial screening identified 52 studies, and 21 additional studies were identified through complementary searches, resulting in 73 studies in the final analysis.

**Summary of findings:**

Climate change is a key factor affecting disease patterns, with pollen seasons starting 10–40 days earlier and yearly emissions increasing by up to 200%. Regarding pathogenesis, early-life rhinovirus C infections with IgE sensitization significantly increase asthma risk (HR = 4.06), while severe asthma shows 40–84% eosinophilic patterns, depending on the assessment approach. On the diagnostic front, advances include multiplex platforms, proteomic biomarkers, and microRNAs. Therapeutically, innovations encompass biologics combined with allergen immunotherapy, nanobody-based therapeutics, and microbiota interventions.

**Conclusions:**

These developments point toward personalized management of respiratory allergic diseases. However, challenges remain in research with underrepresented populations and accessibility. Moving forward, the key priority is integrating this diverse knowledge into practical strategies that advance precision medicine in respiratory allergic diseases.

## Introduction

Respiratory allergic diseases are showing epidemiological changes driven by the interaction of genetic predisposition, environmental exposures, urbanization effects, and other sociodemographic determinants (1). This evolution raises important questions about how these conditions will manifest in the coming decades, particularly as climate change emerges as a significant factor (2, 3).

Current epidemiological studies reveal diverse patterns, from changes in allergenic plant phenology due to global warming (4) to genetic differences between populations that influence asthma susceptibility (5). Characterization of severe asthma populations shows heterogeneity in inflammatory phenotype distribution, with predominance of allergic inflammation in multiple regions worldwide (6). Predictive models indicate that warmer temperatures will advance spring pollen emission by 10–40 days, prolonging seasons and increasing annual emissions up to 200% (3).

In parallel, advances in diagnostic technologies are providing new tools to better characterize these diseases (7), while the therapeutic landscape undergoes significant changes with treatment options increasingly tailored to each patient's specific mechanisms (8, 9). Both areas are moving quickly, building on what already works while opening doors to approaches that were not possible until recently (10, 11).

**Figure 1:**
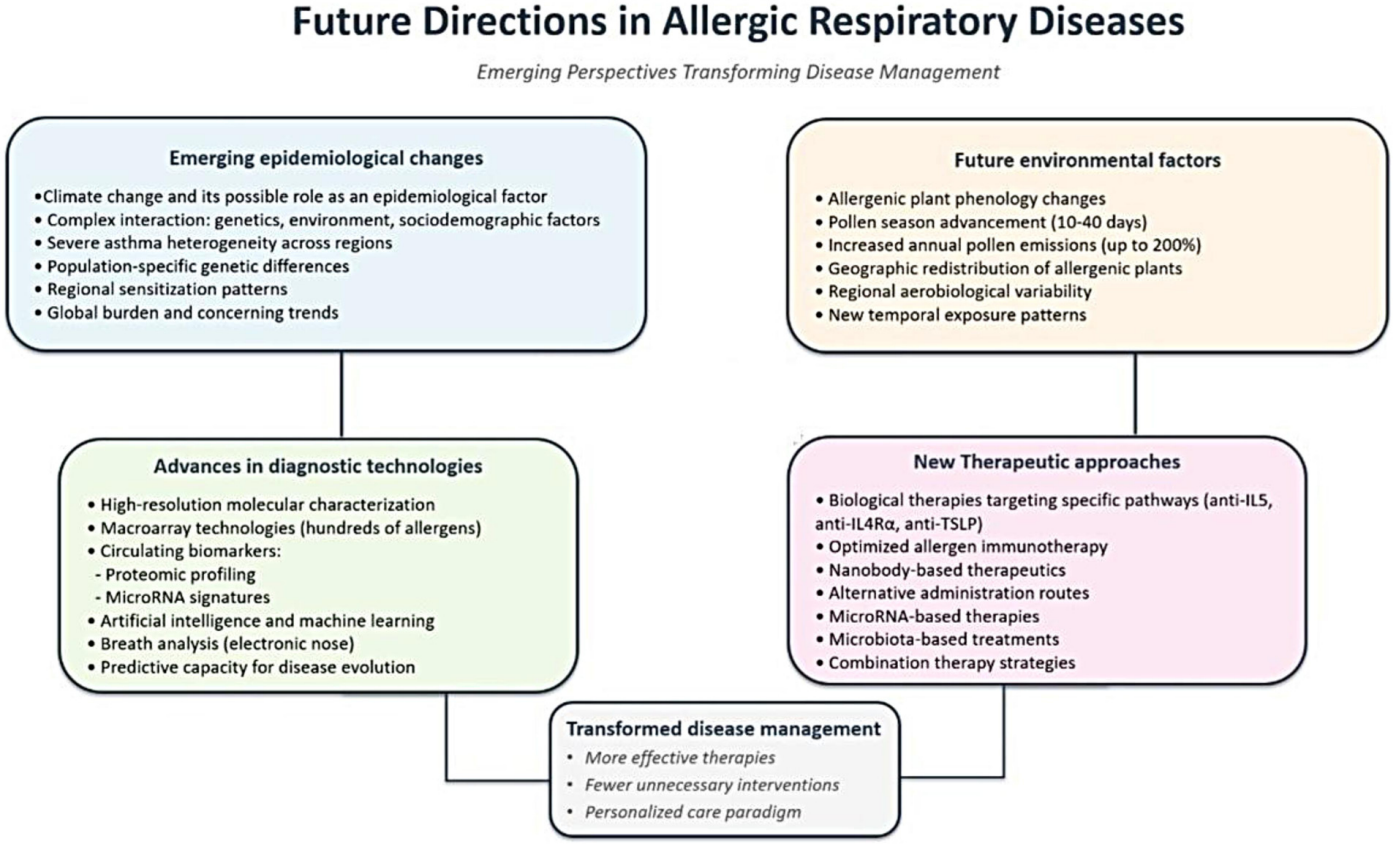
Conceptual framework of future directions in allergic respiratory diseases toward precision medicine. The diagram illustrates four key domains contributing to precision medicine: emerging epidemiological changes, future environmental factors, advances in diagnostic technologies, and new therapeutic approaches, all converging toward transformed disease management.

These developments are pushing respiratory allergy management toward precision medicine. However, the challenge lies in effectively integrating these different dimensions of knowledge to develop strategies that are both scientifically robust and implementable in clinical practice. This review examines emerging epidemiological trends, environmental factors, diagnostic advances, and therapeutic approaches that are transforming disease management toward a personalized care paradigm **(**Figure 1**).**

## Literature search

**Figure 2:**
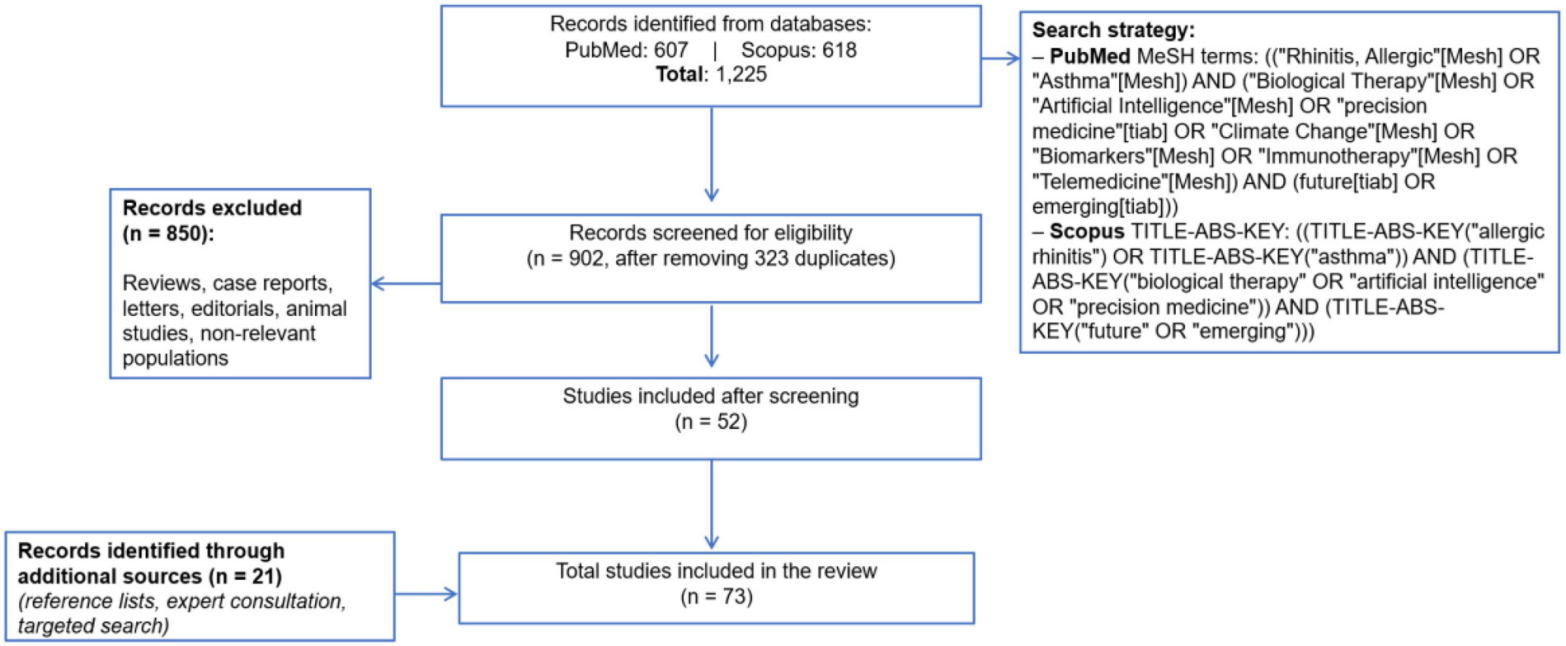
Literature search and study selection flow.

A literature search was conducted in PubMed and Scopus to identify studies addressing future and emerging perspectives in allergic rhinitis and allergic asthma. The search combined terms related to disease management, diagnostic technologies, environmental factors, and therapeutic innovations, and was limited to publications in English and Spanish on human populations published between 2015 and 2025. The complete search strategies are detailed in Figure 2

## Study eligibility, selection, and data extraction

We used a two-phase selection process to identify emerging innovations in the management of allergic respiratory disease. In the first phase, automatic filtering was applied to exclude publication types that would not contribute primary evidence on emerging advances, including all types of reviews (systematic, meta-analyses, narrative, scoping, and umbrella), conference proceedings, letters to the editor, editorials, and case reports. Simultaneously, studies were required to specifically mention terms related to allergic respiratory diseases (“allergic rhinitis”, “allergic asthma”, “atopic”) along with indicators of innovation or future perspective.

In the second phase, thematic relevance and evidence quality were assessed. Studies were required to address human populations with allergic rhinitis and/or allergic asthma, focusing on meaningful advances in treatment, diagnostics, epidemiological patterns, or environmental factors. Only primary studies with original data and consensus documents with prospective perspectives were considered.

Studies were excluded if they involved animal models without direct clinical correlation, populations with conditions too narrowly defined to inform broader clinical practice in allergic rhinitis or asthma, non-allergic rhinitis or asthma, purely historical studies, established treatments without significant innovative components, purely incremental improvements of existing technologies, or experimental studies without evidence of clinical feasibility. This selection approach allowed identification of genuinely transformative advances while maintaining clinical relevance and practical applicability.

After applying all selection criteria, 52 studies were included in the initial review. Subsequently, a complementary targeted search was conducted to ensure inclusion of key studies and recent developments in each of the four thematic areas. This complementary search included: (1) manual review of reference lists from already included studies, (2) specific search for publications from recent years in each thematic area, and (3) verification of key studies frequently cited in specialized literature, and (4) expert-guided inclusion of studies identified through the clinical and research experience of co-authors with specialized expertise in allergy and respiratory diseases. This complementary search enabled the inclusion of 21 additional relevant studies, bringing the total to 73 in the final analysis (Figure 2).

## Emerging epidemiological changes

The epidemiology of respiratory allergic diseases is constantly changing, reflecting multiple factors that influence their development and progression. Recent studies document emerging patterns of interactions among genetic predisposition, demographic shifts, environmental exposures, urbanization and its associated lifestyle changes, and sociodemographic determinants. This epidemiological evolution raises important questions about how these conditions will manifest in the coming decades, with exposome approaches developing as tools to evaluate environmental exposures and their effects on allergic disease development (1).

Climate change appears to be an important epidemiological determinant of respiratory allergic diseases. A long-term Italian epidemiological study analyzing 36,255 individuals during 1957–2006 found that annual asthma incidence correlates with specific climatic patterns, including Summer North Atlantic Oscillation and drought severity index, with a shared average periodicity of 6 years (2). Additionally, research in northwest Tuscany has reported significant associations between daily environmental aeroallergen concentrations and pediatric asthma hospitalizations, with a 10-grain/m^3^ increase in total aeroallergen concentration associated with increased hospital admission risk (OR = 1.054, 95% CI: 1.011–1.098) (12).

Future climate projections indicate modifications in the burden of respiratory allergic diseases at continental levels. Predictive models for the United States suggest that by the end of the century, warmer temperatures will push spring pollen emission 10–40 days earlier and summer and autumn weeds and grasses 5–15 days later, significantly extending the pollen season. Temperature and precipitation alter daily pollen emission peaks by −35 to 40% and increase total annual pollen emission by 16%–40%. Increased atmospheric CO₂ may enhance pollen production, and doubling pollen production alongside climate change could increase end-of-century emissions by up to 200%. These simulations suggest that increased pollen and longer seasons could enhance seasonal allergy probability (3).

However, these predictive models present important limitations that must be considered. Climate studies are primarily based on projections for North America and Europe (3, 4), with scarce representation of tropical and subtropical regions where much of the world's population resides. Furthermore, various studies reveal conflicting patterns regarding the impact of climate on allergic diseases. The Italian research found that asthma rates follow repeating 6-year cycles linked to climate patterns (2), while the US study predicts steady, progressive changes with pollen seasons getting longer each year and emissions continuously increasing (3). These divergent patterns may reflect both methodological differences, since one study measured historical asthma incidence while the other modeled projected pollen emissions, and genuine regional variation in climatic drivers and vegetation composition. Additional confounders such as urbanization, air pollution, and diagnostic practices may further contribute to the observed discrepancy.

Beyond these environmental determinants, early-life viral infections emerge as important factors that could shape asthma development trajectories. A multicenter prospective study of 716 infants hospitalized for bronchiolitis demonstrated virus-specific differences in the risk of recurrent wheeze. Infants with rhinovirus C had a greater risk compared to those with respiratory syncytial virus alone (HR, 1.58; 95% CI, 1.08–2.32). Only infants with rhinovirus C and childhood IgE sensitization had substantially greater risks of recurrent wheeze (HR, 3.03; 95% CI, 1.20–7.61) and asthma development at 4 years (HR, 4.06; 95% CI, 1.17–14.1) (13). A birth cohort followed until 13 years confirmed that rhinovirus illnesses with wheeze during the first 3 years persistently predict asthma until adolescence (OR, 3.3; 95% CI, 1.5–7.1), whereas respiratory syncytial virus infections lose this association over time (14).

Characterization of severe asthma populations reveals considerable variation in inflammatory phenotypes. A multicenter physician survey across Latin American, Eurasian, Middle Eastern, and Chinese countries analyzed 876 patients with severe uncontrolled asthma. Among patients with available laboratory data, 40% had elevated eosinophils (≥150/µL), while 91% had IgE levels of 30–1,500 IU/mL, indicating a predominance of allergy-driven asthma (6). Notably, the International Severe Asthma Registry (ISAR) reported a substantially higher prevalence of eosinophilic asthma, with approximately 84% of patients classified as most likely to have eosinophilic asthma (15). These differences likely reflect variations in patient populations, geographic regions, and phenotyping approaches. Importantly, distinguishing T2-high from T2-low endotypes, characterized respectively by eosinophilia, elevated FeNO, and nasal polyps vs. their absence, is clinically relevant, as this distinction directly informs the selection of targeted therapies (15, 16).

Epidemiological patterns of respiratory allergic diseases reflect the influence of population genetic diversity and regional environmental factors. Multi-trait genetic analysis in East Asian populations identified 52 significant genomic regions associated with asthma, revealing important differences in genetic architecture between European and Asian populations, with genetic diversity analysis in underrepresented groups showing ancestry-specific disease prevalence patterns (17), and specific variants like rs75326924 in the CD36 gene, reducing asthma risk exclusively in East Asians (5) and RORA gene variants influencing type 2 cytokine responses in admixed populations (18).

Allergic sensitization patterns also vary according to regional climatic classifications: a study in 4 Peruvian cities documented differences in Blo t 5 sensitization between temperate arid zones (25.3%) vs. warm zones (6.3%, *p* = 0.050) (19). Additionally, comparative analysis between Lima and Tenerife, regions with similar Köppen climate (BWh), revealed distinct molecular serodominance patterns in 181 patients with rhinitis and/or asthma, with intermediate allergens like Der p 5 and Der p 21 reaching prevalences above 30% (20). In pediatric populations, 82.7% showed aeroallergen sensitization, with *Dermatophagoides farinae* (65.2%) most common, followed by *Dermatophagoides pteronyssinus* (53%) and *Blomia tropicalis* (47.7%) (21). These early sensitization patterns seem to influence long-term disease trajectories.

The temporal evolution of respiratory allergic diseases reveals patterns that help elucidate their natural history. The Italian multicenter study followed 401 patients with seasonal allergic rhinitis for six years, documenting persistence in 93.3% of cases. The condition evolved toward greater complexity, with associated asthma increasing from 36.7% to 48.6% and oral allergy syndrome rising from 23.4% to 37.7% (22). A Swedish prospective study with 2,250 participants (8–19 years) demonstrated 3% annual incidence with marked sex differences: higher incidence in girls (37.4% vs 29.8%) but greater remission in boys (45.4% vs 32.2%). Early sensitization (≤8 years) emerged as the strongest predictor of allergic rhinitis development and reduced remission possibilities, with rates of 63.5% in non-sensitized individuals vs. 30.2% in sensitized individuals (14, 22).

Understanding the global burden of respiratory allergic diseases reveals concerning epidemiological trends requiring urgent attention. A global ecological analysis from 1990 to 2019 documented a median asthma incidence of 402.0 per 100,000 inhabitants, with particularly elevated rates among those under 10 years (1,380.3 per 100,000). This study shows that each 1 °C increase in maximum temperature variability could increase global asthma risk by 5%, with more pronounced effects in high-latitude areas (23). Complementing these findings, a multi-ancestral meta-analysis including 22 biobanks with 153,763 asthma cases and 1,647,022 controls identified 49 previously unreported genetic loci associated with asthma (24). Climate change is intensifying weather events, leading to increases in air pollution, pollen season duration, and pollen allergenicity (25), with direct implications for the onset, exacerbation, and management of childhood allergic asthma (23–26).

These changing patterns highlight the need for approaches that account for multiple factors: climate effects on aeroallergens, genetic differences in susceptibility, specific virus-host interactions that shape disease progression, and the potential for symptoms to evolve over time. Understanding these shifts is crucial because environmental factors are changing rapidly and could reshape allergen exposure patterns over the next few decades. Addressing these shifts will also require expanding research to underrepresented regions, particularly through regional monitoring of aeroallergen dynamics in densely populated low- and middle-income countries where land-use changes and urbanization may act as additional drivers of disease burden. Taken together, these emerging findings move beyond the traditional view of respiratory allergic diseases as conditions driven primarily by allergen exposure, highlighting the complex interplay of climatic, genetic, and early-life factors that will need to be accounted for in future research and clinical practice.

## Future environmental factors

Environmental factors influencing respiratory allergic diseases are undergoing modifications that will alter patterns of recurrent aeroallergen exposure. These environmental changes represent a direct extension of climatic trends that have already been identified as epidemiological determinants.

Plant phenology with allergenic potential is being altered by global warming, resulting in new temporal exposure patterns. A longitudinal study based on Danish herbarium specimens collected over 190 years demonstrated that four specific species (*Poa pratensis, Dactylis glomerata, Festuca rubra, Holcus lanatus*) are flowering significantly earlier in response to warmer spring temperatures (4). Similarly, Australian aerobiological data show latitudinal gradients in timing, duration, and peak number of pollen seasons, suggesting these changes transcend regional boundaries (27). Herbarium data reveal that pollen season duration is extending, and peak pollen loads occur earlier, patterns reflected in biogeographically dependent variation of atmospheric pollen diversity observed in the southern hemisphere (4, 27).

Geographic redistribution of allergenic plants is creating new epidemiological risk maps. Predictive modeling for common ragweed (Ambrosia artemisiifolia) in the eastern United States demonstrated substantial contraction in central Florida, the southern Appalachian mountains, and northeastern Virginia, while northward expansion areas are projected, particularly in the northeast (28). Redistribution patterns are not exclusive to North America, as aerobiological analyses from Australia and New Zealand have revealed biogeographic variation in atmospheric pollen diversity, establishing these geographic changes as a global phenomenon (27). Most ragweed distribution increase is projected for mid-century, with moderation toward 2070, implying sensitivity to climatic variability and creation of specific temporal windows of increased risk (27, 28).

Nevertheless, evidence on the geographic redistribution of allergenic plants remains limited. Studies focus on specific species (primarily *Ambrosia artemisiifolia*) in temperate regions (28), while data on tropical allergenic plants affecting broader populations are lacking. Additionally, redistribution models do not consider anthropogenic factors such as urbanization and land use changes, which may be more determinant than climatic factors in certain regions (27, 28).

Phenological changes, geographic redistribution of allergenic plants, and regional variability in pollen patterns are redefining aeroallergen exposure. As these patterns continue to shift, diagnostic technologies will need to keep pace to properly characterize the exposures patients face.

Urban air pollution represents another major environmental determinant of respiratory allergic diseases, acting synergistically with climate change to amplify disease burden through interconnected biological and epidemiological mechanisms (29). In industrialized and highly urbanized settings, rising concentrations of traffic and industry pollutants, including particulate matter (PM₂.₅ and PM₁₀), nitrogen dioxide (NO₂), ozone (O₃), sulfur dioxide (SO₂), and volatile organic compounds, have been consistently associated with increased prevalence, severity, and exacerbation of allergic rhinitis and asthma (30, 31). Chronic exposure to these pollutants damages airway epithelial integrity, increases mucosal permeability, and facilitates allergen penetration, thereby enhancing interactions with immune cells and amplifying type 2 inflammatory responses (29, 32).

These effects operate through several interconnected mechanisms. Particulate matter and gaseous pollutants activate immune responses via Toll-like receptor pathways (particularly TLR4) (33), oxidative stress from reactive oxygen species, and polycyclic aromatic hydrocarbon sensing through the aryl hydrocarbon receptor (29). These processes induce epithelial barrier damage and promote the release of pro-inflammatory mediators such as IL-6, CXCL8, and GM-CSF, generating a cytokine milieu that amplifies Th2-driven hypersensitivity responses characteristic of allergic diseases, particularly in sensitized individuals with pre-existing IgE-mediated immunity. Both short- and long-term exposure to these pollutants impair mucociliary clearance and facilitate allergen penetration, further intensifying type 2 immune responses (29, 32).

Air pollution alone doesn't directly seem to cause respiratory allergic diseases; however, epidemiological studies consistently link exposure to traffic-related pollution and fine particulate matter (PM₂.₅) (32), with increased asthma incidence, symptom exacerbation, poor disease control, and similar adverse outcomes in allergic rhinitis (29). Moreover, the impact of air pollution appears to be modulated by geographic, socioeconomic, and age-related factors, with children showing greater susceptibility to fine particulate matter. As aeroallergen patterns shift and pollution exposure increases, characterizing these complex molecular signatures will require diagnostic tools capable of capturing individual sensitization profiles at scale, including multiplex allergen platforms, proteomic approaches, and microRNA-based biomarkers (30).

## Advances in diagnostic technologies

Traditional allergy diagnosis has long relied on skin prick tests and single-allergen specific IgE measurements, approaches that provide a useful but incomplete picture of a patient's sensitization profile and offer limited insight into the mechanisms driving disease. Building on these conventional methods, the field has progressively moved toward molecular and multiplex platforms that allow a more comprehensive and mechanistically informed characterization of each patient. Studies analyzing genes, proteins, and metabolites are increasingly used to better understand these diseases (7). The integration of molecular data with clinical characteristics allows for the identification of biomarkers and a more accurate classification of the disease, with latent class analysis confirming the heterogeneity of asthma, such as pediatric asthma (34).

To address clinical implementation challenges of these advances, the development of machine learning-based decision support tools seeks to transform complex data into practical applications for personalized asthma medication selection. Electronic medical records provide a cost-effective alternative for patient profiling when profiling or genotyping all patients is infeasible (35). Multiplex diagnostic platforms are revolutionizing molecular characterization of respiratory allergic diseases by overcoming limitations of traditional uniplex methods, which detect single allergens per test.

Macroarray technologies, such as ALEX (Allergy Explorer), enable the simultaneous detection of specific IgE to hundreds of allergens in a single sample. The evolution of this technology has shown progressive improvements in diagnostic accuracy. ALEX-2 demonstrated improved diagnostic performance compared to traditional methods, with studies showing varying levels of concordance depending on the specific allergens and populations evaluated (36). Evolution toward ALEX-3, based on analysis of nearly 400,000 real-world results, increased allergenic molecules from 178 to 218 while reducing extracts from 117 to 82, progressively substituting variable extracts with defined molecular components to improve diagnostic precision (37).

While macroarray technologies have improved specific IgE detection, molecular characterization goes beyond this to analyze circulating biomarkers that reflect the underlying disease processes. Proteomic technologies are contributing significantly, with mass spectrometry analysis in healthy controls, allergic rhinitis patients, and asthmatics, identifying 18 proteins with differential abundance, revealing key processes like complement activation and extracellular matrix organization, with IL-37 gene expression serving as an inflammation severity marker in comorbid conditions (38) highlighting IGFALS protein as a useful biomarker for differentiating allergic from non-allergic asthma (39) alongside IL-5R*α* expression patterns in nasal polyposis (40).

MicroRNAs are also being evaluated to distinguish well-defined asthma subgroups. Analysis of individuals with allergic and non-allergic asthma demonstrated differential expression of microRNA subsets between groups. Specifically, alterations in miR-155, −146a, −374a, and −145 were observed in allergic asthmatics in response to inhaled corticosteroid use (41). A translational approach combining murine models with human cohorts identified a five-microRNA ratio biomarker signature showing ROC curve area of 0.92 in validation (42). However, both proteomic and microRNA-based approaches share important limitations that temper their clinical applicability: validation cohorts remain small, bioinformatic analyses are complex and resource-intensive, and the transition from research findings to routine clinical practice faces considerable technical and cost-related barriers (39, 42).

Complementing these molecular biomarker approaches, artificial intelligence applications are being developed for clinical prediction and real-time diagnostic support. Machine learning models for predicting asthma control deterioration in children one week prior achieved 71.8% accuracy, 73.8% sensitivity, and 71.4% specificity (43), although short-term prediction models using historical clinical data alone show limited performance (44). Environmental exposure chambers are being validated for standardized allergen challenge protocols (45). Text-based conversational agents using familiar messaging platforms provide personalized risk assessment and management guidance through interactive dialogue, with potential to improve risk self-assessment and asthma self-management alongside validated mobile health applications (46).

Molecular biomarkers also offer predictive capacity for allergic disease evolution and comorbidities, including integrated genomic and molecular scores for childhood asthma risk assessment (47). A multicenter longitudinal study of 401 patients with seasonal allergic rhinitis identified predictive biomarkers such as serum IgE to Phl p 1 for rhinitis persistence, Phl p 5 for rhinitis and asthma persistence, Pru p 3 for asthma onset, and Bet v 1 for oral allergy syndrome persistence (22). Molecular characterization of dust mite allergens established specific prevalence patterns: a German cohort of 722 patients followed until age 20 documented that Der p 2, Der p 1, and Der p 23 reached prevalences greater than 40% (group A), providing a diagnostic framework for molecular panel selection (48).

Breath analysis technologies provide non-invasive methods for asthma phenotyping. Breath profiles measured using electronic nose platforms demonstrated the capacity to classify atopy in pediatric and adult patients, with machine learning models discriminating between atopic and non-atopic participants with ROC curve areas of 0.84 and 0.72 in training and validation sets, respectively (49). Fractional exhaled nitric oxide (FeNO), a marker of airway inflammation, uses three clinical cutoff levels—low (<25 ppb), intermediate (25–50 ppb), and high (>50 ppb)—to guide inhaled corticosteroid therapy adjustments. In patients with aeroallergen sensitization, FeNO-guided management reduced exacerbation rates (adjusted rate ratio = 0.67, 95% CI = 0.49–0.91), demonstrating its value in managing type 2 inflammation (50).

Routine diagnostic methods are also evolving through data mining and molecular validation. A retrospective study in 1,110 children with allergic rhinitis used machine learning algorithms to establish a detection model with AUC values of 0.8512 (51). Separately, a prospective study demonstrated that not total IgE levels, but their variability between measurements, predicts future asthma exacerbations (52).

Together, these diagnostic advances are enabling a shift toward precision medicine, where molecular profiling of individual patients now provides the foundation for the targeted therapeutic strategies discussed in the following section, including endotype-driven biologic selection and emerging interventions tailored to specific disease mechanisms.

## New therapeutic approaches

**Figure 3:**
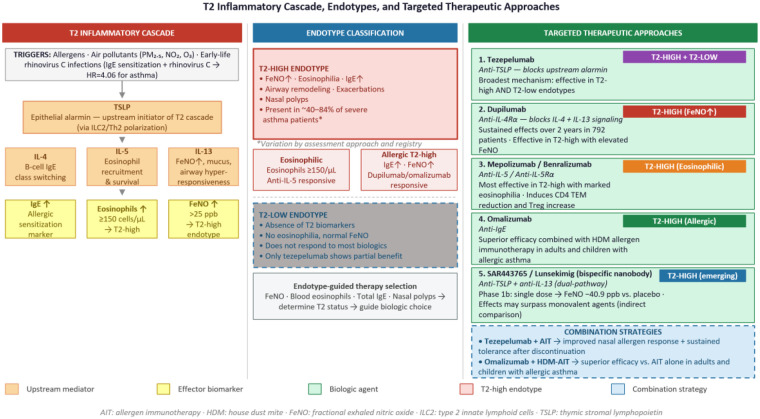
Epithelial release of TSLP in response to allergens, pollutants, and viral infections initiates the T2 inflammatory cascade, driving downstream production of IL-4, IL-5, and IL-13 and generating the characteristic biomarkers of T2-high inflammation: elevated IgE, eosinophilia, and FeNO. This endotype, present in approximately 40–84% of patients with severe asthma, encompasses two partially overlapping subgroups — eosinophilic and allergic T2-high — each with distinct therapeutic implications. In contrast, T2-low patients lack these biomarkers and respond poorly to most biologics, with tezepelumab being the exception given its upstream mechanism of action. Biologic selection is therefore guided by endotype characterization: tezepelumab is effective across both endotypes; dupilumab is preferred when FeNO is elevated; anti-IL-5 agents target marked eosinophilia; and omalizumab is indicated in allergic T2-high disease. Combination approaches pairing biologics with allergen immunotherapy are also represented. AIT, allergen immunotherapy; FeNO, fractional exhaled nitric oxide; HDM, house dust mite; ILC2, type 2 innate lymphoid cells; TSLP, thymic stromal lymphopoietin.

Treatment options for respiratory allergic diseases have evolved considerably toward therapies targeting specific biological mechanisms in each patient. Biological therapies have emerged as the central component of this evolution, with dupilumab demonstrating sustained beneficial effects over 2 years of observation in 792 patients with type 2 inflammation, improving both patient-reported outcomes and objective evaluations (8). This endotype-driven approach is central to precision medicine in severe asthma: anti-IL-5 biologics are most effective in T2-high patients with marked eosinophilia, while agents targeting IL-4 and IL-13 signaling such as dupilumab extend benefit to patients with elevated FeNO but lower eosinophil counts (16) (Figure 3). While the ‘one airway, one disease’ concept underscores the shared inflammatory basis of allergic rhinitis and asthma, the upper and lower airways differ in their local immune microenvironments and therapeutic responses. For instance, tezepelumab combined with allergen immunotherapy primarily improved nasal allergen responses, whereas omalizumab combined with immunotherapy demonstrated efficacy in lower airway outcomes in allergic asthma, suggesting that treatment selection and response assessment may need to account for airway-specific differences (53–55). Simultaneously, new immune regulatory mechanisms are emerging, including the role of sensory neurons in promoting pulmonary immune homeostasis through JAK1 expression in the vagus nerve and in suppressing allergic airway inflammation via CGRP*β*, potentially opening future therapeutic pathways (56).

Recent characterization of anti-IL5 therapy response within the biological therapy arsenal is revealing complex effectiveness patterns extending beyond traditional clinical parameters, with a systematic review confirming efficacy in severe eosinophilic phenotypes (16). Evidence suggests that combining biologics with allergen immunotherapy can enhance therapeutic outcomes beyond those achieved with monotherapy approaches. Combination of tezepelumab (anti-TSLP) with allergen-specific subcutaneous immunotherapy demonstrated superiority compared to immunotherapy alone, improving nasal response to allergen challenge both during treatment and one year after discontinuation, suggesting development of sustained tolerance (53). Similarly, recent randomized controlled trials confirm the superior efficacy of omalizumab combined with house dust mite immunotherapy in adults and children with allergic asthma (54, 55). As these combination approaches move toward routine clinical use, future studies should include cost-effectiveness analyses to assess whether their added benefits can be justified within the economic constraints of real-world healthcare system.

Clinical implementation of these therapies is evolving toward more sophisticated approaches to patient selection and side-effect management. Analysis of 37 experts from 25 European countries revealed substantial differences in severe childhood asthma management, with variation in treatment success evaluation timing, therapy duration, and discontinuation rates (57). Experience with biologics for atopic diseases indicates that response intensity depends on clinical and immunological phenotyping quality, with the emergence of biologics potentially appropriate for multiple simultaneous atopic conditions, raising questions about possible biologic combinations for complex clinical presentations (58).

Beyond these general applications, therapeutic approaches targeting specific molecular pathways are expanding treatment options for refractory conditions. This targeted approach is particularly relevant in patients with NSAID-exacerbated respiratory disease, where additional biological therapy with dupilumab, omalizumab, and mepolizumab showed improvements in subjective and objective parameters after 4 and 12 months (59). NSAID tolerance after biological therapy revealed that omalizumab and dupilumab achieved the highest tolerance frequencies (60% and 40% respectively), while mepolizumab and benralizumab showed lower rates (22% each) (60).

Allergen immunotherapy has emerged as a precision medicine approach that adapts to an individual's specific IgE spectrum and modifies the natural disease course with persistent efficacy after treatment completion (9). Despite therapeutic advances, regulatory obstacles constrain clinical implementation through heterogeneous frameworks that lack specialized guidance for allergenic products (61). The scarcity of standardized reference materials and individualized treatment protocols challenges traditional regulatory paradigms, limiting clinical adoption and patient access to evidence-based approaches (62).

Parallel to these regulatory developments, nanobody-based therapeutics represent an innovative approach to treating respiratory allergies. Preclinical studies demonstrate that nanobodies targeting IL-4R*α* receptors effectively reduce asthma-related biomarkers and airway inflammation in humanized mouse models. Bispecific nanobodies targeting dual pathways, such as IL-4R*α* and IL-5, show greater therapeutic promise than single-target strategies (63, 64). The first clinical example of this technology, known as SAR443765, is a bifunctional nanobody that simultaneously blocks TSLP and IL-13 signaling pathways. In clinical trials, this treatment demonstrated rapid and substantial reductions in FeNO in asthma patients following a single dose, with therapeutic effects that, based on indirect comparisons, may surpass those observed with biological treatments targeting only one of these pathways, though direct comparative trials are needed to confirm this (65).

In addition to these nanobody advances, current research is exploring alternative methods of allergen administration to enhance treatment effectiveness. These new routes include epicutaneous (through the skin), intradermal, intranasal, and intralymphatic administration. The use of immunologically relevant peptides instead of complete allergens is also being investigated as a strategy to develop tolerance more safely (66). Long-term results are encouraging and follow-up studies have demonstrated that clinical efficacy is maintained up to 10 years after treatment discontinuation, especially when treatment duration is at least 3 years (67).

The immunomodulatory effects of these biological therapies are being characterized at cellular and molecular levels. Anti-IL5 treatment induces rebalancing of regulatory and effector T cells in patients with severe asthma, with both medications inducing CD4 TEM reduction and Treg cell increase with different chronologies (68). Complementing these pharmacological approaches, stem cell therapies are being explored as immunomodulatory alternatives, with mesenchymal stem cells transfected with IL-35 showing stronger control of allergic asthma symptoms than stem cells without IL-35 in experimental models (69).

Beyond these cellular approaches, microRNA-based therapies and transcutaneous administration approaches represent innovative directions in therapeutic development. MicroRNA-133b showed beneficial effects on allergic inflammation and symptoms in experimental allergic rhinitis models through modulation of specific inflammatory pathways, reducing both specific IgE levels and characteristic nasal symptoms (10). Within innovative administration modalities, transcutaneous immunotherapy using solid-in-oil nanodispersions loaded with T cell epitope peptides derived from pollen allergens showed suppression of serum IgE antibodies and cytokine production, alleviating allergic symptoms at a therapeutic level similar to subcutaneous injection (70).

Complementing these approaches, research is exploring how different cellular populations can modulate respiratory inflammation. Myeloid-derived suppressor cells show potential for reducing airway inflammation through prostaglandin E2 receptor 4 interactions, with evidence from experimental models suggesting improvements in pulmonary inflammatory response (71).

Growing evidence suggests that gut microbiota influences respiratory immune responses through what has been described as the gut-lung axis, a bidirectional relationship in which intestinal microbial signals shape mucosal immunity at distal sites including the airways (11, 72). Building on this concept, microbiota-based treatments are emerging as strategies for respiratory allergic diseases. Fecal microbiota transplants could alleviate allergic rhinitis through CD4+ T cell modulation via intestinal microbiota restoration, repairing epithelial barrier and modulating CD4+ T cell balance with anti-inflammatory effects through specific cellular signaling pathways like PI3 K/AKT/mTOR and NF-*κ*B (11). This research line is supported by evidence showing how reduced IL-2 response of peripheral blood mononuclear cells exposed to bacteria at 6 months of age associates with elevated total IgE and allergic rhinitis during the first 7 years of life (72).

Optimizing patient selection for these targeted therapies requires advances in diagnostic precision. Deep learning-based approaches for analyzing nasal endoscopy images represent a quantitative alternative to traditional diagnostic methods. These approaches analyze color distribution patterns in the inferior turbinate, combining machine learning classifiers with convolutional neural networks. Current models have achieved diagnostic accuracy above 90% for allergic rhinitis, offering a less invasive and more cost-effective alternative to traditional skin tests and specific IgE measurements (73).

Together, these therapeutic developments reflect a broader shift from empirical to mechanism-driven care, where advances in molecular diagnostics, endotype characterization, and precision biomarkers are now directly informing treatment selection. The convergence of combination biologics, novel administration routes, nanobody-based strategies, and microbiota-based interventions signals that respiratory allergy management is moving toward truly personalized approaches, though their full integration into clinical practice will require addressing persistent challenges in accessibility, cost, and validation across diverse populations.

## Conclusions

Respiratory allergic diseases are evolving in ways that challenge traditional approaches to their management. The complex relationship among genetics, environmental factors, and climate change is creating new disease patterns, while shifts in the timing of allergenic plants’ blooms force us to rethink how we monitor and treat these conditions. While scientific advances offer remarkable opportunities, important limitations remain. Research still focuses heavily on European and North American populations, limiting how broadly findings can be applied. Despite having advanced diagnostic tools, putting them into practice remains difficult due to high costs and limited access, and personalized treatments still need to be tested across more diverse populations worldwide. Expanding access through low-cost diagnostic tools and telemedicine represents a practical step toward precision medicine in settings where the burden of respiratory allergic diseases is high but poorly understood.

These developments point toward matching disease mechanisms directly to treatment choices, offering the promise of more effective and targeted therapies. The challenge ahead is bringing together this growing body of knowledge into strategies that are scientifically grounded and realistic to apply across different healthcare systems around the world.
